# First imported relapse case of *Plasmodium vivax* malaria and analysis of its origin by CSP sequencing in Henan Province, China

**DOI:** 10.1186/1475-2875-13-448

**Published:** 2014-11-21

**Authors:** Ying Liu, Hong-wei Zhang, Rui-min Zhou, Cheng-yun Yang, Dan Qian, Yu-ling Zhao, Bian-li Xu

**Affiliations:** Henan Center for Disease Control and Prevention, Zhengzhou, 450016 China

**Keywords:** *Plasmodium vivax*, Relapse, Imported, CSP

## Abstract

In recent years, there has been a substantial increase of imported *Plasmodium vivax* incidence in Henan Province. As China is in a pre-elimination phase, the surveillance of imported malaria is essential, but there is no good way to distinguish imported cases from indigenous cases. This paper reports a case of a 39-year-old man who acquired *P. vivax* while staying in Indonesia for one month in 2013, and relapsed in Henan, China in 2014. This was diagnosed as vivax malaria based on rapid diagnostic test, Giemsa-stained peripheral blood smear and *Plasmodium* species-specific nested PCR. The genetic sequence for the circumsporozoite protein genes was analysed and the genetic variations were compared with a previously constructed database of Chinese isolates. The results from the circumsporozoite protein (CSP) gene sequence analysis centered on the repeat patterns showed that the imported cases had completely different sequences from any subtypes from Chinese isolates, but well matched with the countries travelled by the patient. The imported vivax cases were able to clearly distinguish from the indigenous vivax cases by detecting the CSP gene and were able to confim its origin by genotyping.

## Background

Malaria is a parasitic infection caused by *Plasmodium falciparum, Plasmodium vivax, Plasmodium malariae* and *Plasmodium ovale.* Globally, between 135 and 287 million people were infected in 2012, of which over 600,000 died [1]. In China, malaria seems to have been known for over 4,000 years and was identified as one of the top five parasitic diseases that affected seriously the socio-economic development after the establishment of the People’s Republic of China in 1949 [2]. In the early 1950s, a malaria epidemic spread in 1,829 counties, i.e., 70-80% of all counties in China [3].

In Henan Province, malaria has been a major health problem historically. *Plasmodium vivax* was endemic in the whole province, while *P. falciparum* was prevalent in the regions south of 33° latitude north. In the early 1970s, the morbidity reported was 16.9%, the number of the malaria cases was highest in the country [4]. Afterwards, the reported malaria cases have declined dramatically through years of efforts, and no local case was reported by the end of 2012, and all the cases were identified as imported. In order to better protect and promote public health, the Henan government carried out an action plan of malaria elimination as a guidance document in 2010, with a goal to eliminate local malaria transmission by 2015 and malaria completely by 2018 [5]. Therefore, the data from each of the cases were analysed carefully to distinguish whether the case was autochthonous or imported since 2013.

This study aimed at a case of imported *P. vivax* from Southeast Asia, who relapsed three months after leaving there, this case was diagnosed by light microscopy, rapid diagnostic test(RDT), nested PCR and sequence analysis. It was the first imported relapse case recorded in Henan Province.

## Case presentation

### Clinical history and laboratory findings

During the work of anti-relapse treatment in 2014, a malaria patient was met by chance, who had a malaria history last year. The patient was a 39-year-old Chinese man with a one-day history of fever (38.1°C), shaking chills and headache; he reported no other constitutional symptoms and without underlying diseases.

**Figure 1:**
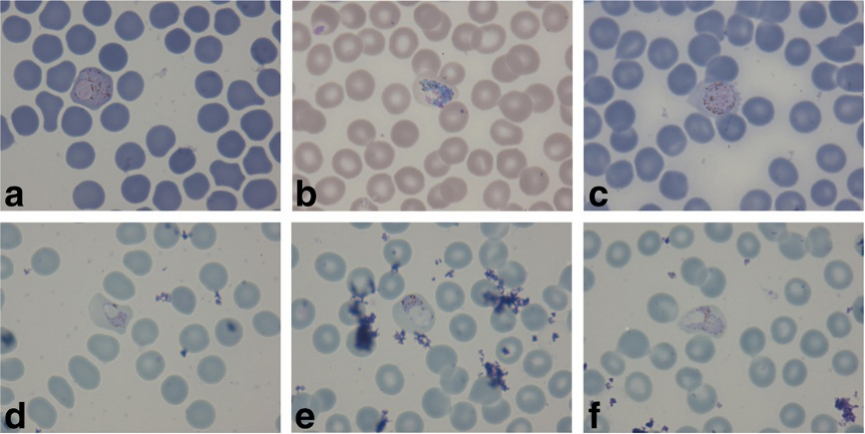
**Subject’s malaria smear.** Giemsa-stained peripheral blood smear at the time of presentation (×1,000) in the primary infection **(a, b, c)** and in the relapse **(d, e, f)**.

At the time of his illness, the patient had lived for about four months in Lankao, which has not been a malaria endemic area in Henan Province. However, before getting back to Lankao, he had been a trucker for one month in Indonesia, a malaria-endemic country. During his stay in Indonesia, he did not have any symptoms related to malaria and did not receive any treatment for malaria. But when he had just come back on 2^nd^ December 2013, he suffered a sudden onset of rigors. He was diagnosed with malaria on 6^th^ December 2013. This case was microscopically identified to be *P. vivax* (Figure 1), the number of malarial parasites per 200 WBCs with a microscope at × 1,000 was counted, assuming 8,000 WBCs per microlitre, and the densities of malarial parasites for the patient were 330/μl. He was treated with a full course of dihydroartemisinin and piperaquine phosphate tablets and primaquine. He did not travel to any other malaria endemic areas after that.

The patient was thoroughly examined. The RDT (Wondfo Biotech Co., Ltd, China) for malarial antigen was positive. The peripheral blood sample showed *P. vivax* malaria by microscopic examination of Giemsa-stained thick and thin blood films; this diagnosis was confirmed at Henan CDC by microscopy experts (Figure 1). The densities of malarial parasites for the patient were 5,760/μl.

The patient was treated with dihydroartemisinin and piperaquine phosphate tablets (immediate dose of 800 mg, followed by 400 mg at 6, 12, 18, and 24 hr) and primaquine (22.5 mg/day for 8 days). He had a rapid and favourable response, his symptoms began to subside after one day of treatment, on the 10th day, the parasites were not observed.

### *Plasmodium*species-specific nested PCR

For the 18S rRNA, nested PCR was performed. DNA was extracted from the patient’s blood samples with the QIAamp DNA Mini kit (QIAGEN Inc, German) according to the manufacturer’s instructions and stored at -20°C. *Plasmodium* genus-specific set (rPLU1/rPLU5) was used for the first-round PCR. Other four pairs of species-specific primers (rFAL1/rFAL3 for *P. falciparum*, rVIV1/ rVIV2 for *P. vivax*, rMAL1/rMAL2 for *P. malariae*, and rOVA3/ rOVA4 for *P. ovale*) that had been reported in a previous study were used in the second-round PCR [6]. Nested PCR was performed using a DNA thermal cycler (iCycler iq-5, Bio-Rad Laboratories, Hercules, California, USA). Primers were provided by Sangon Biotech Co. Ltd, China. Thermal cycling was carried out under the following conditions: initial denaturation at 94°C for 5 min, annealing at 58°C for 30 sec, and extension at 72°C for 60s (34 cycles), and final extension at 72°C for 5 min (30 cycles). The four previously diagnosed clinical samples (*P. falciparum, P. vivax, P. malariae, P. ovale*) and pure water was used as the positive and negative controls, respectively. The amplified DNA product corresponded to *P. vivax* (120 bp). The presence of *P. vivax* in the patient’s specimen was confirmed by nested PCR.

### Genotyping analysis

**Figure 2:**
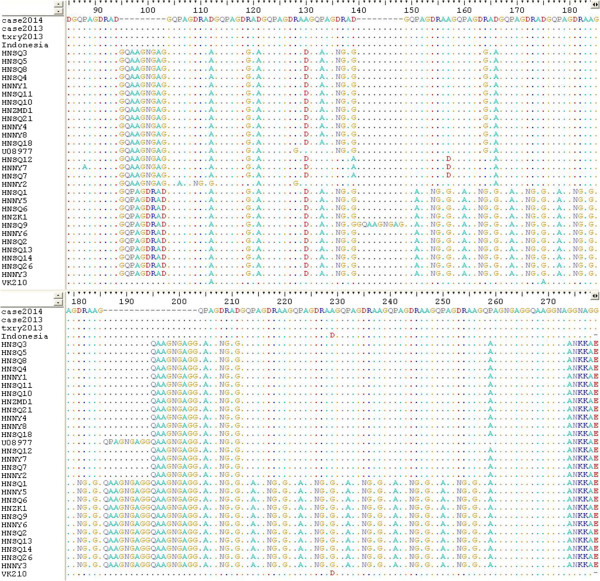
**Amino acid sequence comparison of CSP from** ***P. vivax.*** Amino acid sequences previously found in Chinese *P. vivax* isolates, determined in the present study and from Indonesia were analyzed. The Genbank database accession numbers are as follows: Indonesia (AFI80542 the sequence was collected in 2003 in Borneo Indonesia), U08977(the sequence was collected in 1994 in Guizhou, China), VK210 (M28746). HNZK1, HNZMD1, HNNY1-8, HNSQ1-14, 18, 21, 26 were collected in 2011 in Henan,China. Case 2013 and txry2013 were from the present study.

The polymorphic regions of the circumsporozoite protein (CSP) genes from the DNA samples were amplified and sequenced. The primers and amplification conditions were described by Imwong M *et al.* [7]. The CSP genes from the case and other relative sequences were analysed: three sequences from the National Centre for Biotechnology Information, 27 sequences from previous studies [8], one sequence from the patient’s previous infection (Case 2013), another sequence from his follow-up (txry 2013). It has been found that the sequence from the case (case 2014) nearly had the same CSP sequence as ‘Case2013’ and ‘txry2013’, its sequence was also similar to the Indonesian isolate (AFI80542) with small modification of the repeat number (the case was 18, the Indonesian isolate was 17), but was different from any of the Chinese isolates (Figure 2).

**Figure 3:**
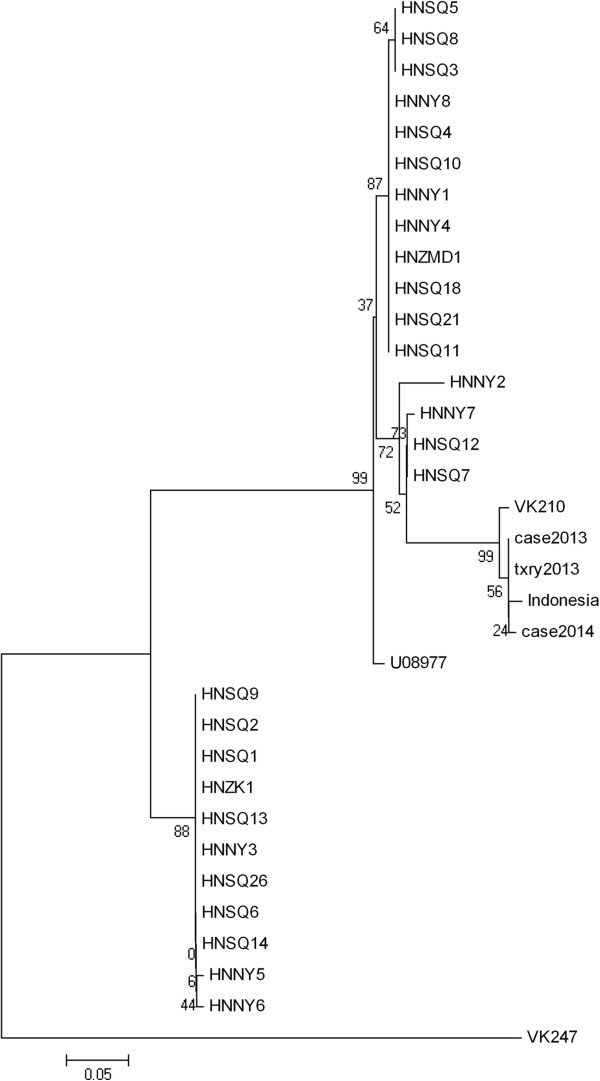
**Neighbour-joining tree of CSP from** ***P. vivax.*** Subtree including the patient’s strain and depicting its phylogenetic relationship with other strains (VK210: M28746, VK247: M28745). Bullets represent >75% bootstrap value (1,000 replicates).

Multiple sequence alignment of all the genes sequences and loci was performed with the algorithm Clustal X software. Phylogenetic trees were constructed using the Molecular Evolutionary Genetics Analysis(MEGA) 4.0 and their reliability was tested by bootstrapping analysis (1,000 replicates). One cluster was considered significant if it was present in more than 75% of the permuted trees (Figure 3).

## Discussion

The case with a history of *P. vivax* infection, had fever, chills, headache for one day, but no other constitutional symptoms. Meanwhile, patient’s current residence, Lankao is a non-endemic area for malaria. Only considering his travel history and historical exposure to *P. vivax*, given the related examination of malaria, the result was positive. This case suggested it was necessary to obtain the epidemiological history when formulating a diagnosis and it also highlighted the importance of considering more etiologies for febrile illnesses, which was especially important in the current era of globalization and increasing residential mobility [9].

This was the first report of a relapse *P. vivax* case from an endemic area in Henan Province. Malaria relapse refers to the re-emergence of the malarial paroxysm after several weeks to more than one year without malarial parasites infection when the parasitemia in the erythrocytic stage has been eliminated at the first onset of malarial patients, which is possiblly related to activated hypnozoites. Relapsable malaria includes *vivax* and *ovale* malaria and other monkey malaria. This case reminded everyone, when facing a patient with fever of unknown origin, obtaining a thorough long-term residential and the history of the spread of malaria is very essential.

The patient’s primary attack of malaria began on 2^nd^ December 2013, he was diagnosed with vivax malaria on the fifth day, the densities of malarial parasites were 330/μl at that time. Then he suffered a relapse on 23^th^ March, was diagnosed on the second day, but the densities of malarial parasites were 5760/μl. The case also showed us, the patients maybe had more tolerance to the malarial parasites when they were suffering a relapse. So in the relapse patients, the disease would develop more rapidly than those with primary infection.

CSP is found in all the mature malaria parasites, which forms a dense coat on the sporozoite’s surface [10]. It comprises three distinct domains: a central repetitive domain with a variable number of tandem repeats, and 2 highly conserved non-repetitive terminals. The central repetitive domain from the CSP varies in sequence and length among *Plasmodium spp* [11]. The *P. vivax* VK210 strain has CSP amino sequence that includes a GDRAA/DGQPA repeat [12, 13]. A variant form, VK247, later identified in Thailand in 1989, possesses an ANGAGNQPG amino acid repeat in the central region [14]. This polymorphic marker was useful for genetic epidemiological surveys where *P. vivax* is endemic.

According to the previous studies, the amino acid sequence of this case in the CSP regions belonged to the VK210 type [15, 16]. For further analysis, the sequence from the case was identical to the sequence he had before (Case 2013) and the sequence from his follow–up (txry2013). At the same time, it showed similarity to the isolate from Indonesia (AFI80542) where the patient had recently travelled, but was different from any of the Chinese isolates. Phylogenetic analysis in the CSP region showed that the investigated strain was related with the strains from Indonesia.

## Conclusions

In this particular case, the patient had been in Indonesia for one month about half year before and the clinical symptoms were not specific, however, malarial parasites were observed in the peripheral blood smear, the nested PCR also supported the result. Considering the test results and his travel history, it could be guessed that he was infected with *P. vivax* during his stay in Indonesia rather than in China. Therefore, careful history taking, including the current residency and travel history, is important to make the diagnosis of malaria. Genotyping is a useful tool to determine the origin of vivax malaria and discriminate imported cases from autochthonous cases. It is very significant in the elimination era.

## Consent

Written informed consent was obtained from the patient for the publication of this report and any accompanying images.
